# A Holistic Analysis of Team Dynamics Using Relational Coordination as the Measure regarding Student Athlete Total Load: A Cross-Sectional Study

**DOI:** 10.3390/sports11050104

**Published:** 2023-05-13

**Authors:** Cathrine Nyhus Hagum, Espen Tønnessen, Marie Aarrestad Nesse, Shaher A. I. Shalfawi

**Affiliations:** 1 Department of Education and Sports Science, University of Stavanger, 4036 Stavanger, Norway; 2 Faculty of Health Sciences, Kristiania University College, 0107 Oslo, Norway

**Keywords:** relational coordination, student athlete management, student athlete wellbeing

## Abstract

Background: Despite its small population, Norway wins a disproportionately large number of medals in international competitions. Therefore, it has been thought that the Norwegian sports model and sports school programs are influential in developing young Norwegian athletes to achieve such results. Today, more than 110 Norwegian private and public schools offer the elite sports program in Norway. Most student athletes attending those schools combine their high school education with elite sports, where they attend training sessions at both school and clubs. The number of people involved with the student athlete on a daily basis (i.e., other student athletes, club coaches, school coaches, schoolteachers, parents, and health personnel) indicate the importance of optimal communication and coordination. However, to the authors’ knowledge, no previous studies have explored communication and coordination among this population group. Therefore, the primary objective of this study was to use a holistic analysis of team dynamics using the Relational Coordination Survey as a measure to explore the relational coordination within and between student athletes, club coaches, and school coaches. A secondary objective of this study was to explore student athletes’, club coaches’, and school coaches’ relational coordination with schoolteachers, parents, and health personnel. In addition, the study aimed to explore differences in student athletes’ relational coordination with their significant others according to sport, school, performance level, sex, and school year. Methods: The quality of relational coordination was measured by a cross-sectional questionnaire of student athletes (*n* = 345), club coaches (*n* = 42), and school coaches (*n* = 25) concerning training load and life load. Multiple one-way analyses of variance were used to assess differences between groups. Results: The results show that student athletes, club coaches, and school coaches perceived moderate to weak relational coordination with parents, schoolteachers, and health personnel. Student athletes’ relational coordination score with parents was the only strong score observed. Furthermore, the results reveal notable differences in student athletes’ relational coordination with the roles according to their characteristics. Conclusions: The findings suggest a potential for enhancing relationships and communication within and between the significant roles involved with student athletes. The results further indicate that those involved with the student athlete should consider a holistic approach to enhance communication and coordination, including physical, psychological, and other life factors, for optimal student athlete management and development. More resources are necessary to facilitate effective communication and coordination regarding the student athlete’s total load.

## 1. Introduction

Despite its small population, Norway wins a disproportionately large number of medals in international competitions [1,2,3,4]. In Norway, the Norwegian sports model and sports school programs are considered influential in developing young athletes [5,6,7]. Since 1981, when the first private Norwegian elite sports school was established, student athletes have had the opportunity to combine high school education with elite sports [6]. Over the last few years, many of Norway’s best individual and team sport athletes have attended elite sports schools, which offer proper facilities and highly qualified coaches. In 2006, the Norwegian national curriculum introduced elite sports as an optional subject in public schools [6,7,8,9]. Today, more than 110 private and public schools offer the elite sports program [10], one of Norway’s most popular programs among high school students [8]. Although differences exist between the programs offered by private and public schools [5,10], a fundamental similarity is that student athletes in the “Elite Sport” program will likely experience a considerable increase in physiological (i.e., training load) and psychological (i.e., stress associated with academic demands, social commitments, employment, and sports participation) loads after enrolment [11,12,13]. Additionally, most Norwegian high schools keep competitive sports and education separate [14], and the majority of student athletes will also participate in club training sessions in the evening, in addition to training during school hours. Hence, multiple people are involved with and influence the student athlete’s progression (e.g., club coaches, school coaches, schoolteachers, parents, health personnel, and peers). Therefore, it could be expected that effective communication and coordination dynamics within and between the people involved with the student athlete are of high importance to ensure optimal training load management, foster athletic and academic development, and prevent adverse outcomes [15,16,17,18,19,20]. For example, effective communication and coordination concerning training, schoolwork, and other life demands is essential to ensure sufficient recovery and reduce the risk of injury [11,12,21,22,23]. However, previous research has indicated that the level of coordination and communication between student athletes, schools, and sports clubs varies considerably and depends on local conditions and circumstances [10,11]. Effective communication strategies are critical to put the student athlete at the centre of a holistic, well-rounded development program [24,25].

The effectiveness of communication and coordination and its importance has been proposed in several theories, including Team Dynamics Theory (TDT) and the holistic ecological approach (HEA). Suppose we assume that the people involved with the student athlete and the student athlete themselves are a team. In that case, TDT aims to explain part of the variability in team dynamics and predict team outcomes [26]. The theory involves four inputs: (1) cohesion, which historically has been regarded as a vital variable when studying small-group dynamics [27,28,29]; (2) team mental models [30]; (3) coordination [31,32,33,34,35,36,37]; and (4) collective efficacy [38]. Team Dynamics Theory focuses on the team, with the inter-relationship between individuals as the measurement approach. Therefore, cohesion, team mental models, coordination, and collective efficacy are processes at the team level.

On the other hand, the HEA is built around two working models: (1) the athletic talent development environment (ATDE) and (2) the model of environmental success factors (ESF) [5]. The HEA, with its two working models, has shown its value as a lens to aid the study of a specific environment in talent development [39,40,41]. The dual-career development environment (DCDE) working model is based on the original ATDE working model, where the main change is a revision of the environmental domain [42]. The model illustrates, at the micro-level, that student athletes are at the centre and surrounded by those closest to them (i.e., study peers, family, friends, teachers, and sports coaches). The DCDE considers sports, studies, and private life as domains in student athletes’ development. The sport domain involves the part of the student athletes’ environment directly connected to the sport, the study domain represents elements related to their school activities, and private life refers to the other areas of the student athletes’ lives.

The Relational Coordination Survey (RCS) is a proposed measure used to address team dynamics using a holistic analysis approach [43]. Relational coordination (RC) theory was developed by Jody Hoffer Gittell in the early 1990s from an in-depth field study of flight departures in the airline industry [43]. The theory’s core construct is “a mutually reinforcing process of interaction between communication and relationships carried out for the purpose of task integration” [44]. The theory suggests that the high-quality relationships of shared goals, shared knowledge, and mutual respect contribute to the support of frequent, timely, accurate, and problem-solving communication, thereby allowing key stakeholders to coordinate their work effectively across boundaries. The opposite effect is expected with low-quality relationships, weakening the quality of communication, and hampering stakeholders’ ability to effectively coordinate their work [45]. The network approach to measuring RC involves separately measuring each dyadic tie in a work process. Instead of asking a respondent to evaluate the quality of their communication and relationships with all roles globally, respondents are asked to separately evaluate each of the key roles involved in the work process. This enhances the accuracy of the measurement compared to a global assessment. Furthermore, by assessing each tie separately, one can differentiate the strength of ties within and between different roles in the work process. As a result, it is possible to diagnose which ties are the weakest, and where it may be necessary to intervene to increase the strength of RC [45].

Hence, the primary objective of this study was to use a holistic analysis of team dynamics using the RCS as a measure to explore perceived RC regarding total load (i.e., training load and life load) within and between student athletes, club coaches, and school coaches [43]. A secondary aim was to explore student athletes’, club coaches’, and school coaches’ perceived RC with schoolteachers, parents, and health personnel. In addition, the study aimed to explore differences in student athletes’ perceived RC with their coaches and significant others according to the type of sport, school, performance level, sex, and school year. To the author’s knowledge, this is the first study investigating RC in a sports context.

## 2. Materials and Methods

### 2.1. Study Design

**Figure 1:**
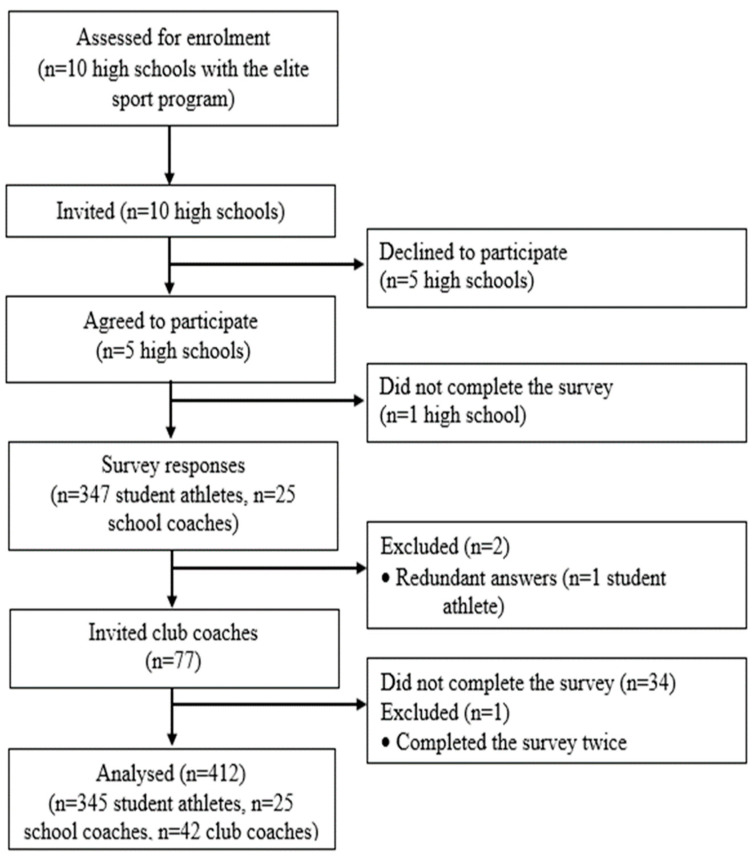
Participant flow throughout the study.

The study employed a cross-sectional design. All Norwegian high schools in a selected county offering the optional school subject “Elite Sport” were given equal opportunity to participate (n = 10; 2 private, 8 public). Student athletes born between 2004 and 2006 and enrolled in the elite sport program were eligible for inclusion. The school coaches and club coaches included in the study were connected to one or more of the included student athletes. Five high schools agreed to participate (1 private, 4 public). Figure 1 shows the participant flow.

### 2.2. Sample Size

In accordance with Statistics Norway (SSB, www.ssb.no, accessed on 24 January 2023), the total number of student athletes attending a sports program in Norwegian high schools in 2020 was measured at 12,547. The sample size was calculated using the online Raosoft sample size calculator (Raosoft, Inc., 2004, http://www.raosoft.com/samplesize.html, accessed on 29 January 2023). With a confidence level of 95%, a margin of error of 5%, and a response distribution of 50%, the recommended sample size was 373.

### 2.3. Participants

The participants in the study were 412 respondents, including student athletes enrolled in the elite sport program (n = 345; 84%), club coaches (n = 42; 10%), and school coaches (n = 25; 6%). The student athletes were involved in 23 different sports, where football (43%), handball (20%), ice hockey (6%), swimming (5%), and cycling (4%) were the most frequently reported sports. Descriptive statistics of the participants are presented in Table 1. Informed consent was obtained from all participants. The study was approved by the Norwegian Social Science Data Services (NSD) (project number 836079).

### 2.4. Instrument

The validated RCS [46,47] was first used in the Nine-Hospital Study of Surgical Patients [48] and has since then been used in numerous different contexts, including the commercial, education, health care, and human service sectors [43].

**Figure 2:**
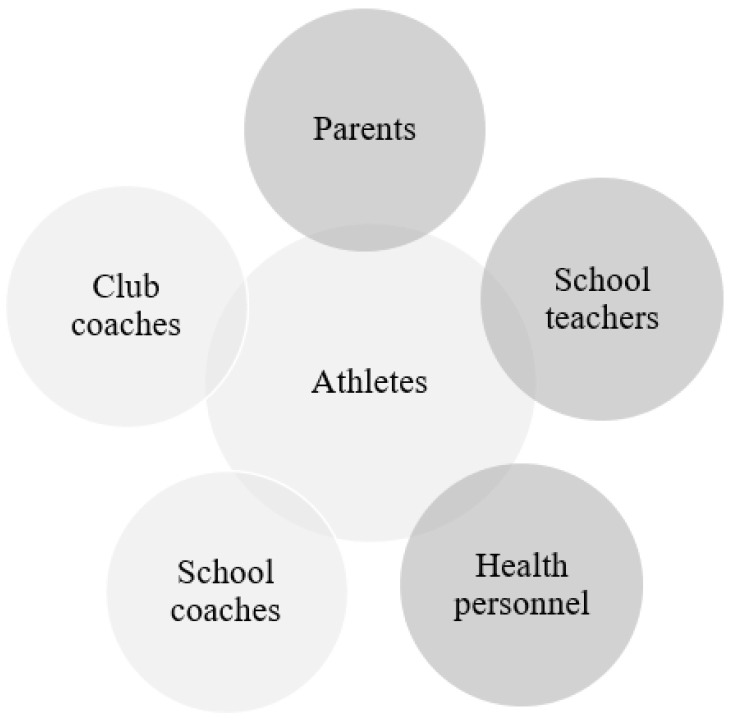
The included roles engaged in student athletes’ training load, performance development, and life load (*light grey was surveyed, whereas dark grey was not surveyed*).

The RCS consists of two factors: communication and relationship. Communication consists of four items (frequent communication, timely communication, accurate communication, and problem-solving communication), whereas relationship consists of three items (shared goals, shared knowledge, and mutual respect). The items are answered on a 5-point Likert scale (Supplementary Materials, Table S1). In addition to the response options 1 through 5, a “not applicable” option was included to allow respondents to indicate that RC with a particular role was not needed. These answers were recoded as missing values [49]. Respondents were asked to complete each item according to their perception of communication or relationships with specific roles included in the study (i.e., student athletes, parents, schoolteachers, school coaches, club coaches, and health personnel). Figure 2 illustrates the included roles engaged in student athletes’ training load, performance development, and life load.

The RCS was previously translated from the original English to Norwegian by Hustoft et al. [50]. A psychometric assessment of the Norwegian version of the RCS suggested a two-factor solution with a Cronbach’s alpha (α) of 0.93 and 0.80 for communication and relationship factors, respectively [50]. We used the version from Hustoft et al. [50] as a guide when changing the wording in the survey so that it would be appropriate to our setting.

### 2.5. Data Collection

Survey data were collected between February and April 2020. By using SurveyXact version 8.0 [51], the questions from the RCS were manually added to the program. In addition, we included background questions regarding age, sports experience, sex, school year, type of school, training volume, school program, type of sport, and performance level. Student athletes were asked to evaluate their current performance level with the following question: “In your opinion, how would you rate your performance level compared to other peers in the same sport in Norway, where the top 1% is the best in your sport?” For the analysis, responses were dichotomised into above the top 5%, top 5–25%, top 25–50%, or below the top 50%. Three different roles were surveyed, and participants from each group received a questionnaire formulated for student athletes, school coaches, or club coaches. The questionnaires were tested by distributing a link electronically to two independent persons. First, the questionnaire targeting student athletes was distributed electronically to the schools that agreed to participate in the study. The Head of Department further distributed the questionnaire to the student athletes during an allocated teaching hour. During the data collection, investigators were present at the school to answer any potential questions. The questionnaire targeting school coaches was distributed to them personally. Finally, club coaches were contacted for participation in the study based on the responses from the student athletes (e.g., which sports club they belonged to and their performance level).

### 2.6. Statistical Analysis

All analyses were carried out using IBM SPSS statistics version 27.0 (IBM Corporation, Armonk, NY, USA) and Mplus version 8.4 (Muthén and Muthén, Los Angeles, CA, USA). Descriptive statistics are presented as the mean (M) and standard deviation of the mean (SD) or frequencies. First, responses for the item “frequent communication” were re-coded such that 1 = “far too little”, 2 = “far too much”, 3 = “too little”, 4 = “too much”, and 5 = “just right” [49]. Then, preliminary analyses investigating the normal distribution were conducted (Table 2). Skewness and kurtosis were examined, and the Kolmogorov–Smirnov test (KS), the Shapiro–Wilk test (SW), and a multivariate normality test were conducted. Skewness and kurtosis values between ±1.0 were considered excellent, and values in the range of ±1.0–2.0 were considered acceptable [52]. For the KS, SW, and the multivariate normality test, a *p*-value of >0.05 was used to indicate normally distributed data [53].

An exploratory factor analysis (EFA) was conducted to test the construct validity of the RSC [54]. We used the goemin (oblique) rotation and a maximum likelihood estimator (MLR), considering the multivariate non-normality in the measures (Table 2). The number of factors was determined based on the eigenvalues, the scree plot, and the parallel analysis [55]. Model fit indices were not considered, as growing evidence indicates that it is inappropriate to use model fit indices to select the number of factors in a scale evaluation framework [56]. According to Kaiser’s rule, the number of eigenvalues ≥1 would represent unique factors [55]. In the scree plot, the number of factors above the elbow would indicate the optimal number of factors. For the parallel analysis, the factor should be retained when the average eigenvalues from the random data were smaller than the reported eigenvalues for the EFA [57]. McDonald’s omega (ω) with confidence intervals (CIs) was calculated to estimate scale reliability. A value of ≥0.70 was considered acceptable [58,59], and a maximal estimate of 0.90 was determined regarding redundant items [52,60]. Cut-off points for weak, moderate, and strong RC ties within and between roles are based on norms from previously collected RC scores collected between 2012 and 2015 (Table 3) [45].

To investigate the difference in perceived RC between the surveyed roles (i.e., student athletes, club coaches, and school coaches), multiple one-way analyses of variance (ANOVAs) were conducted. In addition, multiple one-way ANOVAs were conducted to investigate the difference in student athletes’ perceived RC according to the type of sport (individual or team), school (public sports-friendly high school or private elite sport high school), performance level (above the top 5%, top 5–25%, top 25–50%, or below the top 50%), sex (female or male), and school year (first, second, or third year). A Bonferroni adjustment was applied to correct for multiple comparisons and reduce the likelihood of Type I error [61,62]. Partial eta squared (η_p_^2^) was used to determine the effect size and was interpreted as 0.01 = small, 0.06 = medium, or 0.14 = large [63].

## 3. Results

### 3.1. Exploratory Factor Analysis

**Figure 3:**
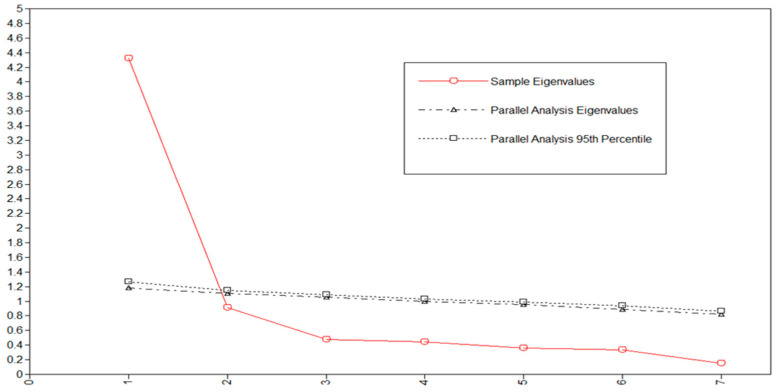
Scree plot showing the data-based and parallel-analysis-based eigenvalues.

The one-factor solution was preferred based on analyses of eigenvalues (Table 4) and the scree plot (Figure 3) containing data-based and parallel-analysis-based eigenvalues. Table 5 shows the factor loadings, residual variances, and the calculated McDonald’s ω. All items had high factor loadings in the one-factor solution (0.627–0.903). The factor also constituted high reliability with a McDonald’s ω of 0.892 (95% CI 0.876–0.919). The estimated unexplained residual variances (i.e., uniqueness) ranged from 0.184 to 0.607. Hence, the results reveal that the RCS has good construct validity and high reliability.

### 3.2. The Strength of Perceived RC

**Figure 4:**
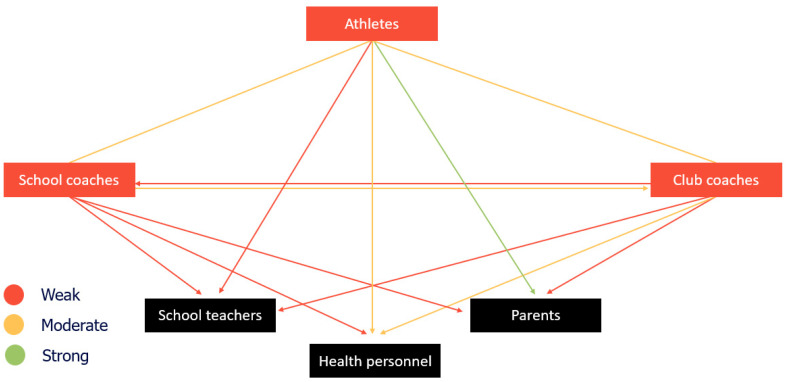
The quality of relational coordination among the participants. Note: Black boxes indicate roles that were not surveyed. Arrows from one box to another indicate the perceived quality of relational coordination between the roles. Lines between two boxes indicate a mutual quality of relational coordination between the roles.

The mean values of RC with the roles included in the present study are presented in Table 6. Figure 4 is based on the information from Table 6 and illustrates RC among the roles according to the cut-off points from Gittell (2018) (Table 3).

### 3.3. Differences in Perceived RC between Roles

The one-way ANOVA results with descriptive statistics and effect sizes are presented in Table 7. No marked differences were observed in student athletes’, school coaches’, or club coaches’ perceived RC with club coaches or health personnel (*p* > 0.05). However, the results indicate notable differences in student athletes’, school coaches’, and club coaches’ perceived RC with school coaches (*p* < 0.001), schoolteachers (*p* < 0.001), parents (*p* < 0.001), and student athletes (*p* < 0.001). Post hoc tests with Bonferroni adjustment indicated marked differences between student athletes’ and club coaches’ RC with school coaches (M difference = 0.99, *p* < 0.001) and between school coaches’ and club coaches’ RC with school coaches (M difference = 1.27, *p* < 0.001). Furthermore, notable differences were found between student athletes’ and club coaches’ RC with schoolteachers (M difference = 1.05, *p* < 0.001) and between school coaches’ and club coaches’ RC with schoolteachers (M difference = 1.31, *p* < 0.001). In addition, there were marked differences between student athletes’ and club coaches’ RC with parents (M difference = 0.77, *p* < 0.001) and between student athletes’ and school coaches’ RC with parents (M difference = 0.77, *p* < 0.001). Lastly, the results indicate notable differences between student athletes’ and school coaches’ RC with student athletes (M difference = −0.63, *p* = 0.002) and between student athletes’ and club coaches’ RC with student athletes (M difference = −0.45, *p* = 0.005).

### 3.4. Student Athletes’ Perceived RC According to Characteristics

The one-way ANOVA results with descriptive statistics and effect sizes are presented in Table 8. For the type of sport, there was a notable difference between team sport student athletes’ and individual sport student athletes’ RC with club coaches (M difference = −0.36), school coaches (M difference = −0.33), schoolteachers (M difference = −0.40), parents (M difference = −0.37), and health personnel (M difference = −0.52). No marked differences in perceived RC with the different roles were found for the type of school. Regarding performance level, there was a notable difference in perceived RC between student athletes based on performance level (i.e., above the top 5%, top 5–25%, top 25–50%, and below the top 50%) with parents (*p* = 0.048). No marked differences in perceived RC with club coaches, school coaches, schoolteachers and health personnel were found between student athletes of the four performance-level categories. There was a marked difference in perceived RC with parents between the performance-level categories. However, when examining multiple comparisons with Bonferroni adjustment, there was no marked difference in RC between the student athletes of the four performance-level categories. With regard to sex, no notable differences were found between female and male student athletes’ perceived RC with club coaches, school coaches, schoolteachers, parents, or health personnel. Lastly, the results regarding the school year indicated no notable difference in RC with club coaches, school coaches, schoolteachers, or health personnel. There was a marked difference in first-, second-, and third-year student athletes’ perceived RC with parents. Post hoc tests with Bonferroni adjustment indicated a marked difference in RC with parents between first- and second-year student athletes (M difference = 0.28, *p* = 0.012).

## 4. Discussion

The purpose of the present investigation was to use a holistic analysis of team dynamics using RCS as a measure to explore perceived RC within and between student athletes, club coaches, and school coaches. A secondary aim was to explore student athletes, club coaches, and school coaches’ perceived RC with schoolteachers, parents, and health personnel. In addition, the study aimed to explore differences in student athletes’ perceived RC with their coaches and significant others according to the type of sport, school, performance level, sex, and school year. The main finding from this investigation was that the RC level between the surveyed roles (i.e., student athletes, school coaches, and club coaches) was moderate to weak. Furthermore, student athletes, club coaches, and school coaches perceived a moderate to weak RC with parents, schoolteachers, and health personnel. The only strong RC present was student athletes’ RC with parents. The results also revealed notable differences in student athletes’ RC with the roles (i.e., club coaches, school coaches, schoolteachers, parents, and health personnel) according to their characteristics.

### 4.1. Perceived RC between the Student Athlete, Club Coach, and School Coach

The results from this investigation indicate that the RC ties between and within the student athletes, school coaches, and club coaches were either moderate or weak (Figure 4). As shown in Table 7, student athletes and school coaches perceive a moderate RC with club coaches. Furthermore, student athletes perceive a moderate RC with school coaches, while club coaches perceive a weak RC with school coaches. Lastly, school and club coaches perceive a moderate RC with student athletes. These results suggest a potential for enhancing team dynamics between and within these roles to meet the minimum optimal RC score (i.e., between RC = >4.0 and within RC = >4.6). It is well known that the relationships between those involved in the student athlete’s training are key to their development and sporting success [15,16,17]. In addition, according to the RC theory, high-quality relationships of shared knowledge, goals, and mutual respect reinforce and are reinforced by frequent, timely, accurate, and problem-solving communication, resulting in effective coordination [43]. Therefore, student athletes, school coaches and club coaches should strive to develop high-quality relationships. However, relationships of low quality undermine effective communication, hindering successful coordination [43], and potentially impairing the student athlete’s academic and sporting development. According to Jowett [64], viewing coaching as centred around the coach–student athlete relationship, in which coaches and student athletes are meaningfully connected, can promote mutually empowering inclusivity. Such meaningful partnerships can also function as a tool that motivates, guarantees, pleases, and supports well-being, performance, and experiences [65]. Implementing the correct communication strategies (i.e., support, motivation, and conflict management strategies) can influence the athlete–coach relationship positively, resulting in a higher degree of athlete training satisfaction, individual treatment, and performance [66,67,68,69]. Hence, a good starting point for achieving effective team dynamics is to initiate regular informal and formal communications (i.e., meetings) between the roles, educate to enhance competence, and utilize electronic diaries for relevant roles.

### 4.2. Perceived RC from Student Athletes, School Coaches, and Club Coaches with Parents

As shown in Table 7, student athletes perceive a notably better RC with parents compared to club coaches and school coaches. As illustrated in Figure 4, the RC tie from student athletes to parents was the only strong tie in the present investigation. This finding implies that student athletes perceive high-quality relationships and communication with their parents, which can facilitate effective coordination regarding their total load [43]. It is well-established in the literature that parental involvement and support play a vital role in the youth sports experience and in performance and skill development [70,71,72,73,74,75]. For example, parents’ behaviours can strongly influence a student athlete’s motivational characteristics in sports, such as perceived competence, enjoyment, enthusiasm, and intrinsic motivation [76,77]. According to Smoll et al. [78], parents are inextricably involved in the youth sports experience. Hence, they are essential roles at the micro-level and have the potential to impact the quality of the experience for all involved roles. Fostering positive parental involvement and strengthening the relationship between parents and coaches can therefore generate beneficial outcomes. Research has shown that poor communication, mistrust, and a lack of shared goals between parents and coaches compromises student athletes’ development [79]. In the present investigation, we do not have data regarding parents’ perceived RC with the other roles. This limits our ability to generate a coherent picture of the mutual relationships between the roles, especially the parent–coach relationship. However, several guidelines for communicating and working with parents in youth sports have been proposed [73,78,80,81].

### 4.3. Perceived RC from Student Athletes, Club Coaches, and School Coaches with Schoolteachers

Figure 4 illustrates that student athletes, club coaches, and school coaches perceive weak RC with schoolteachers. However, although the strength of the relationship was considered weak with all the surveyed roles, Table 7 shows that student athletes and school coaches perceive a notably stronger RC with schoolteachers than with club coaches. A possible explanation for this is that school coaches and schoolteachers work in the same location, perhaps making communication easier. School coaches and schoolteachers must adhere to the curriculum, making it difficult to coordinate all their activities with sports clubs. The interaction between school and club can lead to conflict when both want maximal endeavour from the student athlete [82]. Previous research has suggested that formal and informal communication can be helpful in the coordination of activities between the club, school, and sports association [83]. Hence, when coaches create training plans it is essential to consider information from the schoolteachers, so that during periods with increased schoolwork the training load can be adequately reduced, and vice versa.

Research shows that burnout and drop-out from sports are frequently linked to non-training-related stressors. As such, a holistic analysis approach based on a conscious decision about the acceptable overall load on the student athlete was advised [84]. Strengthening communication and coordination regarding the student athletes’ total load, within and between roles at both the micro and macro-level, is necessary to ensure optimal athlete wellbeing and reduce the risk of injury [11,12,21,22,23]. For instance, one can measure both external and internal load to obtain an overview of the student athletes’ training status and training load [85]. Furthermore, to reveal physiological and psychological training-related stress, one can use weekly subjective self-report measures such as the Multicomponent Training Distress Scale [86,87]. In addition, to capture the student athlete’s general life stress, one can use the Adolescent Stress Questionnaire monthly [88,89]. These measures have previously been used in combination, when individualised sport-specific training programs were given weekly to student athletes transitioning to a sports academy high school [90].

### 4.4. Student Athletes, Club Coaches, and School Coaches Perceived RC with Health Personnel

As shown in Table 7, there were no marked differences in perceived RC with health personnel between student athletes, school coaches, and club coaches. Perceived RC with health personnel will likely vary according to the student athlete’s health status. It is reasonable to assume that injured student athletes and their respective roles communicate more with health personnel than non-injured student athletes. Previous research has indicated that the quality of communication between the medical team and the coach is associated with injury burden and player availability in elite football [23]. In addition, a previous injury is a leading intrinsic risk factor for sustaining a new injury [91,92,93]. Hence, and due to the high injury prevalence in student athletes enrolled in elite sports schools [94,95], enhancing the relationship dynamics between health personnel and coaches may facilitate faster and better injury diagnosis, benefit the rehabilitation process, and contribute to more robust student athletes returning to sport post injury [96,97]. Monitoring athletes’ training load and implementing strategic recovery periods can not only reduce injury risk, but also maximise performance [20].

### 4.5. Student Athletes’ Perceived RC with the Roles According to Their Characteristics

#### 4.5.1. Type of Sport

As shown in Table 8, student athletes from individual sports perceive markedly higher RC with all roles compared with team student athletes. The effect size was small to moderate. Previous research suggests that it is often more challenging to facilitate relationship dynamics between the federation, club and region in team sports compared with individual sports [18]. It is reasonable to assume that it is easier for student athletes from individual sports to communicate and coordinate factors influencing their total load (e.g., physical training, competitions, schoolwork, and general life stress) compared with team sport athletes. In individual sports, coaches can focus more on managing and optimising load for a single athlete, rather than having a whole team of players to consider. The findings in the present investigation correspond with research from Rhind et al. [69], indicating that student athletes from individual sports report being closer and more committed to their coach. In addition, student athletes in individual sports believed that their coach felt more respect, trust, and appreciation for them compared to team student athletes, likely due to interacting more frequently on a one-to-one basis [69]. The reason why individual student athletes perceived stronger RC with their parents than team student athletes are unknown. Previous research has suggested that student athletes with resourceful parents, in combination with physiological advantages (e.g., puberty stage and growth), manage the increase in training and dual workload better [12], which could explain this finding.

#### 4.5.2. Student Athletes’ Performance Level

No notable differences were found in perceived RC with any of the roles between student athletes of different performance levels (Table 8). However, Table 6 indicates that student athletes performing in the top 5% perceive a strong RC with club coaches, while lower performing athletes perceive only a moderate RC with club coaches (Table 6). Furthermore, Table 6 shows that the strength of RC is reduced with lower performance level for both club coaches and school coaches. Findings from Berntsen and Kristiansen [98] indicate an obvious endorsement misfit between student athletes participating in sports “for fun”, and their coaches with a “work hard” mentality which undermines the student athletes’ need-satisfaction, commitment, performance, and well-being. Successful coaching in the elite sport school context requires coherence between the aims of the coach and the aims of the student athlete [98]. A possible explanation for the findings in the present study could be that student athletes at the highest performance level have shared goals with their coaches, more so than student athletes of lower performance levels. If the student athlete, club coach, and school coach have a shared goal of performing at the highest level it is more likely that they will achieve effective coordination dynamics regarding the student athletes’ total load to meet this goal.

#### 4.5.3. The Type of School

We did not find a notable difference in student athletes’ perceived RC with the roles according to school type (i.e., private elite sports school or public sports-friendly school). In contrast, a recent study of football players and their coaches found that the close integration of the school and club settings in elite sports schools enables better communication dynamics regarding the overall workload compared to less structured sports-friendly schools [10]. There are several possible explanations for these contradictory findings. First, our results are based on a number of different individual and team sports, and not exclusively football. Second, we used a quantitative method and collected data from both sexes within three school years. Third, the data were collected from a larger sample and in another Norwegian county. Lastly, coach experience and qualifications may have a role to play in how coaches communicate with their student athletes [99]. These factors may influence the student athlete’s perceived RC regarding training load and general life stress with the essential roles around them, further highlighting the importance of context.

#### 4.5.4. School Year

We did not find marked differences in perceived RC with club coaches, school coaches, schoolteachers, or health personnel between first-, second-, or third-year student athletes. In light of TDT [26], every team has a start and end point. It would therefore be reasonable to assume that relationships between the roles at the micro-level would become more robust over time due to regular meetings, potentially fostering suitable conditions for better communication and coordination dynamics. Our results indicate that first-year student athletes perceived a stronger RC with parents than second-year student athletes. The effect size was small to moderate. Within the dual-career pathway, and especially in the transitions involved, student athletes might face challenges and stressors in sports (e.g., pressure to train and perform well, and increased training loads) and education (e.g., attending classes, completing assignments, and passing exams) [100]. That the perceived RC is strongest among first-year student athletes is a positive finding, since the challenges they face may be more substantial during transition periods (e.g., transitioning to a sports high school).

## 5. Conclusions

Perceived RC between student athletes, school coaches, and club coaches was moderate to weak. Furthermore, student athletes, club coaches, and school coaches perceived a moderate to weak RC with parents, schoolteachers, and health personnel. The only strong RC present was student athletes’ RC with parents. The results also revealed notable differences in student athletes’ RC with the roles according to their characteristics.

The findings presented in this study offer several important practical implications. First, there is a need for the different roles to strengthen their relationships and communication to achieve effective team dynamics regarding student athletes’ total load. This can be accomplished through regular informal and formal meetings, education to enhance competence, and by using electronic diaries available for the relevant roles. Educating student athletes and encouraging them to monitor and register their training, lifestyle, competitive performances, and psychological aspects may help in the early identification of an overtrained or stressed state [101].

However, many student athletes might experience self-report measures as an additional burden [85]. Consequently, such measures should be incorporated into theoretical sessions during school hours. Teachers and coaches should highlight the value of such measures by facilitating an understanding of training loads and the implications for attendance, performance, and health [84]. Involving the student athlete when designing training plans can provide a significant developmental and educational opportunity [102]. At the micro-level, the importance of talking to the student athletes should not be undervalued, in order to better understand how individual student athletes are tolerating and responding to the training [85]. In addition, a partnership between student athletes and the roles should be developed at the micro and macro-level to ensure purposeful, accurate and valuable data collection relevant to the individual’s sport, while also considering less burdensome data collection methods [85]. The combination of regular conversations and student athlete self-report measures can potentially strengthen the shared knowledge between the student athletes and the involved roles, facilitating a higher degree of team dynamics [43]. Managing data from training diaries and questionnaires is time-consuming and requires extra resources in the school or club. Employing qualified persons responsible for student athlete monitoring who are able to pass on information to relevant roles connected to the student athlete could enhance communication and coordination dynamics within and between the roles at the micro-level. Increased communication and coordination dynamics concerning the student athletes’ total load can hopefully improve team outcomes, increase motivation, reduce student athlete drop-out rates, and promote optimal sporting and academic development.

### Limitations and Future Research

Although the current study provides a number of valuable insights, some limitations must be acknowledged. First, only student athletes from one Norwegian county were included, limiting generalisability to different cultures and countries. Second, we did not record the duration of the relationships of the included roles, which could have impacted the results. Third, we used a cross-sectional design to measure perceived RC at a given point in time. A longitudinal research design, where relationship quality is measured over time, would provide valuable information. Fourth, only three roles within the student athlete environment were surveyed (student athletes, school coaches, and club coaches). Future research should collect data from all roles involved with the student athlete, giving a more complete picture of the mutual relationships between the roles. That said, roles within the macro-level, such as regional and national clubs and sports associations, could also be included in further research. The study would also have been more informative if it had included interviews with those who had the strongest RC scores. By doing this, it would be possible to identify concrete measures leading to strong perceived RC. In the future, a mixed-method design could yield valuable insights, by first utilising the RCS and subsequently interviewing and observing high-RC environments. In this way one could gain an in-depth understanding of how relationship quality is conceptualised across separate dyadic connections and what different roles believe are the critical elements of their relationships with other groups [103].

## Figures and Tables

**Table 1:** Descriptive statistics of the 412 participants in the study.

Characteristics	Modalities	M ± SD or Frequency		
Role		Athletes (*n* = 345)	Club coaches (*n* = 42)	School coaches (*n* = 25)
Age		17.15 ± 0.94	38.15 ± 12.27	40.44 ± 8.41
Sports experience in years (2) ^1^		11.08 ± 2.56		
Sex	Female	147	10	4
	Male	198	32	21
School year	First year	142		
	Second year	95		
	Third year	108		
Training volume (4)	Sports-friendly programme	13.88 ± 3.74		
	Elite sport programme	15.45 ± 4.84		
School program ^2^	Specialisation in general studies	204		
	Sports and physical education	141		
Type of sport (2)	Individual	98	8	
	Team sport	245	34	
Performance level	Top 1–5%	18	1	
	Top 5–25%	159	9	
	Top 25–50%	153	24	
	<Top 50%	15	8	

Note. M = mean; SD = standard deviation. ^1^ Values in brackets indicate missing values for athletes. ^2^ Student athletes attending a specialisation in general studies have chosen “Elite Sport” as an optional program subject. Student athletes attending sports and physical education have, in addition to the optional program subject “Elite Sport”, theoretical and practical subjects related to sports (i.e., physical activity, sports science, training management, and sports and society).

**Table 2:** Descriptive statistics of the items and tests of normality.

Item	N	M	SD	Skewness	Kurtosis	KS (*p*)	SW (*p*)
Frequent communication	411	4.1	0.8	−1.1	1.3	0.000	0.000
Timely communication	408	3.0	0.8	0.2	0.0	0.000	0.001
Accurate communication	408	2.9	0.9	0.2	0.1	0.000	0.000
Problem-solving communication	403	3.5	1.0	−0.2	−0.6	0.004	0.000
Shared goals	403	3.5	0.8	−0.1	−0.4	0.000	0.000
Shared knowledge	409	3.2	0.7	−0.1	0.1	0.020	0.124
Mutual respect	407	3.8	0.9	−0.4	−0.2	0.000	0.000

Note. M = mean; SD = standard deviation; KS = Kolmogorov–Smirnov test; SW = Shapiro–Wilk test.

**Table 3:** Cut off points for weak, moderate, and strong relational coordination ties.

Strength	Within Roles	Between Roles
Weak	<4.1	<3.5
Moderate	4.1–4.6	3.5–4.0
Strong	>4.6	>4.0

Note. The cut off point is from Gittell (2018).

**Table 4:** Eigenvalues for sample correlation matrix.

Factor	Eigenvalue
1	4.32
2	0.91
3	0.48
4	0.44
5	0.36
6	0.33
7	0.15

**Table 5:** Geomin rotated loadings, McDonald’s omega (ω), and residual variances for the one-factor solution.

	One-Factor Solution	
Item	1	Residual Variances
Frequent communication	0.627 *	0.607
Timely communication	0.903 *	0.184
Accurate communication	0.889 *	0.210
Problem-solving communication	0.705 *	0.502
Shared goals	0.677 *	0.542
Shared knowledge	0.686 *	0.530
Mutual respect	0.649 *	0.579
McDonald’s ω (95% CI)	0.892 (0.876–0.919)	

Note. * Significant at the 5% level.

**Table 6:** Mean values of perceived relational coordination within and between the roles.

		Rating of								
		CC	SC	A Top > 5%	A Top 5–25%	A Top 25–50%	A < Top 50%	ST	P	HP
**Ratings by**	CC	3.7	2.6	3.8	3.6	3.6	3.1	1.9	3.3	3.7
	SC	3.6	3.9	4.1	3.9	3.6	3.5	3.2	3.3	3.3
	A top 1–5%	4.0	3.8	3.5	3.5	3.5	3.3	3.1	4.3	3.8
	A top 5–25%	3.7	3.7	3.0	3.2	3.0	2.9	2.9	4.1	3.6
	A top 25–50%	3.5	3.5	2.9	3.12	3.0	2.6	2.9	3.9	3.4
	A < top 50%	3.8	3.7	3.2	3.5	3.5	3.5	3.5	4.1	3.6
	All	3.7	3.5	3.4	3.5	3.3	3.1	2.9	3.8	3.6

Note. CC = club coaches; SC = school coaches; ST = school teachers; P = parents; HP = health personnel; A = athletes.

**Table 7:** One-way ANOVA results with descriptive statistics and effect sizes.

RC with	Role	N	M	SD	95% CI		*p*	η_p_^2^
					LB	UB		
Club coach	Athlete	337	3.64	0.95	3.54	3.74	0.875	0.00
	School coach	24	3.61	0.86	3.25	3.98		
	Club coach	40	3.71	0.84	3.45	3.98		
School coach	Athlete	341	3.60	0.85	3.51	3.69	<0.001	0.11
	School coach	25	3.89	0.71	3.59	4.18		
	Club coach	38	2.62	1.05	2.27	2.96		
School teacher	Athlete	327	2.96	1.02	2.85	3.07	<0.001	0.09
	School coach	23	3.22	0.65	2.94	3.50		
	Club coach	31	1.90	0.66	1.66	2.15		
Parents	Athlete	345	4.05	0.73	3.97	4.13	<0.001	0.13
	School coach	25	3.28	0.71	2.99	3.57		
	Club coach	39	3.28	0.71	3.05	3.51		
Health personnel	Athlete	298	3.52	0.98	3.41	3.63	0.310	0.01
	School coach	21	3.27	1.08	2.77	3.76		
	Club coach	38	3.67	0.86	3.39	3.96		
Athlete	Athlete	295	3.15	0.89	3.04	3.25	<0.001	0.05
	School coach	24	3.78	0.65	3.50	4.05		
	Club coach	42	3.59	0.71	3.37	3.81		

Note. LB = lower bound of 95% confidence interval; UB = upper bound of 95% confidence interval; η_p_^2^ = partial eta squared.

**Table 8:** Multiple comparisons of athlete’s perceived RC according to the type of sport, performance level, sex, and school year.

RC by Type of Sport								
		N	M	SD	95% CI		*p*	η_p_^2^
					LB	UB		
Club coach	Team	240	3.54	0.92	3.42	3.66	0.002	0.03
	Individual	95	3.90	0.99	3.70	4.10		
School coach	Team	243	3.51	0.85	3.40	3.62	0.001	0.03
	Individual	96	3.84	0.82	3.67	4.00		
Schoolteacher	Team	232	2.84	1.00	2.71	2.97	0.002	0.03
	Individual	93	3.24	1.04	3.02	3.45		
Parents	Team	245	3.95	0.75	3.86	4.05	<0.001	0.05
	Individual	98	4.32	0.62	4.19	4.44		
Health personnel	Team	213	3.38	0.95	3.25	3.51	<0.001	0.06
	Individual	83	3.90	0.97	3.69	4.11		
**RC by Type of school**								
Club coach	Sports-friendly	240	3.59	0.96	3.47	3.71	0.177	0.01
	Elite school	97	3.75	0.93	3.56	3.94		
School coach	Sports-friendly	243	3.58	0.86	3.47	3.69	0.474	0.00
	Elite school	98	3.65	0.85	3.48	3.82		
Schoolteacher	Sports-friendly	235	2.96	1.02	2.83	3.09	0.999	0.00
	Elite school	92	2.96	1.05	2.74	3.17		
Parents	Sports-friendly	246	4.05	0.71	3.96	4.14	0.918	0.00
	Elite school	99	4.06	0.79	3.90	4.21		
Health personnel	Sports-friendly	211	3.48	0.97	3.35	3.61	0.254	0.00
	Elite school	87	3.62	0.99	3.41	3.83		
**RC by Performance level**								
Club coach	Top 1–5%	17	3.97	0.73	3.59	4.34	0.149	0.00
	Top 5–25%	156	3.70	1.01	3.54	3.86		
	Top 25–50%	150	3.52	0.92	3.37	3.67		
	<Top 50%	14	3.80	0.74	3.37	4.23		
School coach	Top 1–5%	18	3.82	0.69	3.48	4.17	0.116	0.00
	Top 5–25%	157	3.68	0.88	3.54	3.82		
	Top 25–50%	152	3.48	0.82	3.35	3.61		
	<Top 50%	14	3.69	0.97	3.13	4.25		
Schoolteacher	Top 1–5%	18	3.08	0.93	2.62	3.54	0.248	0.00
	Top 5–25%	149	2.94	1.02	2.77	3.11		
	Top 25–50%	145	2.91	1.05	2.74	3.08		
	<Top 50%	15	3.45	0.89	2.96	3.95		
Parents	Top 1–5%	18	4.30	0.65	3.97	4.62	0.048	0.01
	Top 5–25%	159	4.13	0.70	4.02	4.24		
	Top 25–50%	153	3.94	0.74	3.82	4.05		
	<Top 50%	15	4.06	0.93	3.54	4.57		
Health personnel	Top 1–5%	16	3.79	0.67	3.44	4.15	0.065	0.00
	Top 5–25%	140	3.64	0.98	3.48	3.81		
	Top 25–50%	128	3.35	1.00	3.18	3.53		
	<Top 50%	14	3.55	0.93	3.01	4.08		
**RC by Sex**								
Club coach	Female	145	3.65	0.93	3.50	3.80	0.808	0.00
	Male	192	3.63	0.98	3.49	3.77		
School coach	Female	145	3.63	0.87	3.48	3.77	0.629	0.00
	Male	196	3.58	0.85	3.46	3.70		
Schoolteacher	Female	136	2.99	1.03	2.82	3.17	0.590	0.00
	Male	191	2.93	1.02	2.79	3.08		
Parents	Female	147	3.96	0.79	3.83	4.09	0.054	0.01
	Male	198	4.12	0.68	4.02	4.21		
Health personnel	Female	132	3.53	1.03	3.35	3.71	0.905	0.00
	Male	166	3.52	0.93	3.37	3.66		
**RC by School year**								
Club coach	First year	140	3.71	0.94	3.55	3.87	0.367	0.01
	Second year	93	3.64	0.85	3.47	3.82		
	Third year	104	3.53	1.05	3.33	3.74		
School coach	First year	141	3.72	0.82	3.59	3.86	0.064	0.02
	Second year	92	3.47	0.83	3.29	3.64		
	Third year	108	3.56	0.89	3.39	3.73		
Schoolteacher	First year	133	3.06	1.00	2.89	3.23	0.181	0.01
	Second year	90	2.80	0.97	2.60	3.00		
	Third year	104	2.97	1.10	2.76	3.18		
Parents	First year	142	4.20	0.69	4.08	4.31	0.008	0.03
	Second year	95	3.92	0.68	3.78	4.06		
	Third year	108	3.98	0.81	3.82	4.13		
Health personnel	First year	121	3.66	0.97	3.48	3.83	0.120	0.01
	Second year	82	3.38	0.89	3.19	3.58		
	Third year	95	3.47	1.04	3.26	3.68		
	Elite school	87	3.62	0.99	3.41	3.83		

## Data Availability

Source data will be provided with the Supplementary Materials.

## Supplementary Materials

The following supporting information can be downloaded at https://www.mdpi.com/article/10.3390/sports11050104/s1: Table S1: dataset.
