# Supplementary material for: A Holistic Analysis of Team Dynamics Using Relational Coordination as the Measure regarding Student Athlete Total Load: A Cross-Sectional Study
Source: Sports (Basel). 2023 May 13;11(5):104. doi: 10.3390/sports11050104 (PMC10223519; doi:10.3390/sports11050104)
Supplement: Supplementary file 1 [file sports-11-00104-s001.zip › sports-2387032-supplementary.pdf]

Table 1

The Likert scale in the relational coordination survey

Item	The Likert scale (1 through 5)					
FREQ[1]	Far too little	Too little	Just right	Too much	Far too much	Not relevant
TIME	Never	Rarely	Occasionally	Often	Always	Not relevant
ACCUR	Never	Rarely	Occasionally	Often	Always	Not relevant
PROBL	Never	Rarely	Occasionally	Often	Always	Not relevant
GOAL	Not at all	A little	Somewhat	A lot	Completely	Not relevant
KNOW	Not at all	A little	Somewhat	A lot	Completely	Not relevant
RESP	Not at all	A little	Somewhat	A lot	Completely	Not relevant

Note. FREQ = frequent communication; TIME = timely communication; ACCUR = accurate communication; PROBL = Problem-solving communication; GOAL = Shared goals; KNOW; Shared knowledge; RESP = Mutual respect.

[1]More frequent communication does not indicate higher quality communication. Responses for this question was recoded for analysis such that 1 = "Far too little", 2 = "Far too much", 3 = "Too little", 4 = "Too much", and 5 = "Just right" (Gittell, 2018).
